# TSG-6 released from adipose stem cells-derived small extracellular vesicle protects against spinal cord ischemia reperfusion injury by inhibiting endoplasmic reticulum stress

**DOI:** 10.1186/s13287-022-02963-4

**Published:** 2022-07-13

**Authors:** Xiao Lu, Chengtang Lv, Yuechao Zhao, Yufei Wang, Yao Li, Chengyue Ji, Zhuanghui Wang, Wu Ye, Shunzhi Yu, Jianling Bai, Weihua Cai

**Affiliations:** 1 Department of Orthopedics, The First Affiliated Hospital of Nanjing Medical University, 300 Guangzhou Road, Nanjing, Jiangsu Province China; 2 Department of Orthopaedics, Dongtai Hospital Affiliated to Nantong University, Dongtai City, Jiangsu China; 3 Department of Orthopaedics, Yancheng Third People’s Hospital, Yancheng, 224000 Jiangsu China; 4 Department of Orthopedic Oncology, Changzheng Hospital, Secondary Military Medical University, Shanghai, China; 5 Department of Orthopedic, PLA Navy No.905 Hospital, Secondary Military Medical University, Shanghai, China; 6 Zhejiang University-University of Edinburgh Institute (ZJU-UoE Institute), Haining, Zhejiang China; 7 Department of Orthopedics, Shanghai Tenth People’s Hospital, Tongji University School of Medicine, 301 Yanchang Road, Shanghai, China; 8 Department of Biostatistics, School of Public Health, Nanjing Medical University, Jiangsu Province, Nanjing, 211166 China

**Keywords:** Small extracellular vesicle, Adipose‐derived stem cells, Spinal cord ischemia reperfusion injury, Endoplasmic reticulum stress, Tumor necrosis factor (TNF)-stimulated gene-6

## Abstract

**Background:**

Spinal cord ischemia reperfusion injury (SCIRI) is a complication of aortic aneurysm repair or spinal cord surgery that is associated with permanent neurological deficits. Mesenchymal stem cell (MSC)-derived small extracellular vesicles (sEVs) have been shown to be potential therapeutic options for improving motor functions after SCIRI. Due to their easy access and multi-directional differentiation potential, adipose‐derived stem cells (ADSCs) are preferable for this application. However, the effects of ADSC-derived sEVs (ADSC-sEVs) on SCIRI have not been reported.

**Results:**

We found that ADSC-sEVs inhibited SCIRI-induced neuronal apoptosis, degradation of tight junction proteins and suppressed endoplasmic reticulum (ER) stress. However, in the presence of the ER stress inducer, tunicamycin, its anti-apoptotic and blood–spinal cord barrier (BSCB) protective effects were significantly reversed. We found that ADSC-sEVs contain tumor necrosis factor (TNF)-stimulated gene-6 (TSG-6) whose overexpression inhibited ER stress in vivo by modulating the PI3K/AKT pathway.

**Conclusions:**

ADSC-sEVs inhibit neuronal apoptosis and BSCB disruption in SCIRI by transmitting TSG-6, which suppresses ER stress by modulating the PI3K/AKT pathway.

**Supplementary Information:**

The online version contains supplementary material available at 10.1186/s13287-022-02963-4.

## Introduction

Spinal cord ischemia reperfusion injury (SCIRI) is a devastating complication of aortic aneurysm repair or spinal cord decompression procedures that is associated with neurologic dysfunction and fatal paralysis. Due to limited therapeutic options, SCIRI is a serious threat to human health and quality of life [1, 2]. The pathophysiological mechanisms for SCIRI involve blood–spinal cord barrier (BSCB) disruption, which is a highly specialized brain endothelial structure for maintaining spinal cord homeostasis [3, 4]. Similar to the blood–brain barrier, BSCB is primarily composed of brain endothelial cells connected by tight junction proteins including zonula occludens-1 (ZO-1), claudin-5 and occludin, which limit the entry of peripheral blood cells and plasma components into the spinal cord. Upon damage by ischemia–reperfusion injury, opening of tight junction pathways leads to BSCB disruption, and the neurotoxic products ultimately cause neuronal apoptosis and neurological deficits. Accumulating evidence shows that BSCB disruption and neuronal apoptosis are potential therapeutic targets for SCIRI.

Stem cell-based therapies have been shown to improve motor functions after SCIRI [5–7]. Due to their availability, abundant sources and multidirectional differentiation potential, adipose‐derived stem cells (ADSCs) are the preferred sources for therapeutic stem cells. Indeed, ADSCs have been widely used for the clinical management of a variety of neurological diseases, including spinal cord injuries, ischemic brain injuries and traumatic brain injuries [8–10]. The potential risks of tumor formation and poor cell survival of mesenchymal stem cells (MSCs) that are attributed to immune rejection and ischemia limit the clinical applications of direct stem cell transplantation [11–13]. Transplanted stem cells exert therapeutic effects on the targeted tissues through the paracrine pathway in which the small extracellular vesicles (sEVs) play a crucial role [14]. MSC-derived sEVs are the smallest bilayer nanovesicles, containing various proteins, lipids and regulatory nucleic acids [15–18]. They are released from various cell types and participate in a wide range of intercellular communication and metabolic signaling pathways [19]. Our previous literature studies have demonstrated that sEVs can improve motor function after spinal cord injury [20–24]. In addition, ADSC-derived extracellular vesicles (ADSC-sEVs) exert a therapeutic effect on various central nervous system diseases [25–27]. Thus, we hypothesized that ADSC-derived sEVs can also promote motor function recovery after SCIRI. Tumor necrosis factor-α-stimulated gene-6 (TSG-6) is a multifunctional glycoprotein secreted by mesenchymal stem cells or immune cells in response to inflammation irritation [28]. It was demonstrated that TSG-6 exhibits anti-inflammatory and tissue protective properties across a wide range of disease models [28, 29]. However, whether TSG-6 plays a critical role in SCIRI is still unknown. Endoplasmic reticulum (ER) stress is a central mechanism for the occurrence of neuronal ischemia reperfusion injury [30]. The ER is an essential organelle for responding to cellular stress. However, cellular stress dysregulates normal ER functions. Persistent ER stress inhibits the capacity of the unfolded protein responses (UPRs) to maintain ER homeostasis and, subsequently, leads to cell apoptosis [31]. In addition, bone marrow mesenchymal stem cell-derived sEVs ameliorate intervertebral disk degeneration by inhibiting ER stress [32]. However, it has not been established whether ADSC-sEVs can suppress ER stress in SCIRI.

We show, for the first time, that ADSC-sEVs reduce neuronal apoptosis and inhibit BSCB disruption by suppressing ER stress. Moreover, the role of the candidate protein in ADSC-sEVs-induced modulation of ER stress was assessed. In summary, this study aimed at evaluating the therapeutic effects and underlying mechanisms of ADSC-sEVs in SCIRI.

## Methods

### Isolation and identification of sEVs from ADSCs

Human adipose tissue samples were obtained from consenting female donors. ADSCs were isolated according to a previously reported procedure and grown in Dulbecco’s modified Eagles medium/Nutrient Mixture F-12 (DMEM/F12; Thermo Fisher Scientific, USA) supplemented with 10% fetal bovine serum (FBS; Gibco, USA) [33]. The medium was changed every 3 days, and cells were sub-cultured at a ratio of 1:3 after trypsinization. When the ADSCs reached 80–90% confluence, the culture wells were washed with PBS and the culture medium was replaced with exosome-depleted FBS for additional 48 h. The medium on which ADSCs had been cultured was collected and centrifuged at 300 g for 10 min and at 2000 g for 10 min at 4 °C. The supernatant was sterilized by filtration through a 0.22-μm filter (Steritop, Millipore, USA) to eliminate dead cells and debris. The sterilized supernatant was transferred to an Amicon Ultra-15 Centrifugal Filter (Millipore) and centrifuged at 4000 × g until the volume was reduced to approximately 200 µl. Then, the liquid was layered on a 30% sucrose/D2O cushion and subsequently centrifuged at 100,000 × g for 60 min using an Optima L-100XP Ultracentrifuge (Beckman Coulter). Partially purified sEVs were recovered using an 18 g needle, diluted in PBS, and centrifuged at 4000 × g until the final volume reached 200 µl. The isolated ADSC-sEVs were stored at − 80 °C for further experiments.

The morphology of sEVs was determined using a transmission electron microscope (TEM; Tecnai 12; Philips, Best, The Netherlands). Nanosight tracking analysis (NTA, Nanosight Ltd., Novato, CA) was used to analyze size distribution of sEVs. The specific surface markers of sEVs including CD9, CD63, and CD81 were detected by Western blot.

### Primary cortical neuron culture

Primary cultured cortical neurons were isolated from embryonic day-18 Sprague Dawley (SD) rats as previously reported [34]. Briefly, after isolation of the cerebral cortex and gently removing the meninges, brain cortices were sliced into 1 mm^3^ sections, digested using 0.25% trypsin–EDTA solution (Thermo Fisher Scientific, MA, USA) for 20 min at 37℃ after which the reaction was stopped by adding horse serum (Sigma-Aldrich). Next, cells were collected by centrifugation for 5 min (1000 rmp/min) at 4 °C and resuspended in DMEM/F-12 containing 10% horse serum, glutamine (0.5 mM; Gibco) and 1% penicillin–streptomycin (Thermo Fisher Scientific). After counting, the neurons were seeded on poly-D-lysine-coated plates and the medium was replaced with serum-free neurobasal medium (Thermo Fisher Scientific, USA) supplemented with 2% B27 (Gibco Laboratory, Grand Island, NY), glutamine (0.5 mM; Thermo Fisher Scientific) and 1% penicillin–streptomycin. The culture medium was half-replaced every other day.

### ADSCs-sEVs uptake

Purified ADSC-sEVs were fluorescently labeled using Dil (Molecular Probes, Eugene, OR, USA) according to the manufacturer’s instructions. First, 4 mg/mL Dil solution was added to the PBS (1:200) and incubated for 15 min at 4 °C. The mixture was ultracentrifuged at 100,000 × g for 1 h to remove excess dye from the labeled sEVs. These Dil-labeled sEVs were co-cultured with neurons and bEnd.3 cells for 24 h, and the cells were subsequently washed three times using PBS and fixed in 4% paraformaldehyde. Afterward, ADSC-sEVs uptake was visualized using a confocal microscope. To evaluate sEVs uptake in vivo, DiI-sEVs were intravenously injected into SCI model mice. Mice were anesthetized, and frozen sections were prepared. Then, sections were stained using 4′,6-diamidino-2phenylindole (DAPI) and observed by fluorescence microscopy.

### Overexpression of TSG-6 in ADSCs

To evaluate the functions of TSG-6, ADSCs were transduced with a TSG-6 overexpressed lentiviral vector, while an empty vector construct was used as the negative control for TSG-6. The lentiviral-TSG-6 sequences were: forward, CGCGCTAGCATGATCATCTTAATTTAC, and reserve, CGCACCGGTTTATAAGTGGCTAAATCTT. To perform lentiviral transfection, ADSCs were cultured in lentivirus‐containing medium for 4–6 h at 37 ℃ according to the manufacturer's guidelines. Then, the lentivirus was removed and fresh medium was added to continue cultivating cells for 48 h.

### Oxygen–glucose deprivation and reperfusion (OGD/R) model

The OGD/R model was established to mimic SCIRI in vitro as previously described [35]. Briefly, primary neurons were cultured in sugar-free medium and then placed in an airtight chamber that was flushed with a continuous flux of gas mixture (95% N2/5% CO2) for 15 min. Afterward, the chamber was sealed and placed in a 37 °C incubator for an additional 30 minutes of OGD. Neurons were then maintained in a normal medium under normoxic culture conditions for 12 h. Cultured neurons were incubated with sEVs (6 × 10^8^ particles) for 24 h.

### Apoptosis detection by TUNEL staining and flow cytometry, in vitro

To detect apoptosis following OGD/R injury, neurons or bEnd.3 cells were incubated with terminal deoxynucleotidyl transferase dUTP nick end labeling (TUNEL) solution (Roche, Basel, Switzerland) for 30 min according to the manufacturer’s protocols. After staining with DAPI for 5 min, cells were imaged using a fluorescent microscope. Apoptosis was analyzed using ImageJ (NIH, Bethesda, MD, USA) to calculate the proportion of TUNEL-positive cells.

Cell apoptosis was also detected using an Annexin V-FITC/PI apoptosis detection kit (BD Bioscience, CA, USA). Briefly, 1 × 10^5^ neurons were washed using PBS, stained with 10 μL of Annexin V-fluorescein isothiocyanate (FITC) and 5 μL of propidium iodide (PI) in the dark. Apoptotic cells were detected by flow cytometry (BD Biosciences, MA, USA).

### Spinal cord ischemia–reperfusion injury model

The use of animals in this study was approved by the Ethics Committee of Nanjing Medical University, and all procedures were in accordance with the Guidelines for the Care and Use of Laboratory Animals of the China National Institutes of Health. C57BL/6 mice (male, 20–30 g) were purchased from the Animal Center of Nanjing Medical University (Nanjing, Jiangsu). The SCIRI model was established as previously described [36]. Briefly, mice were anesthetized using pentobarbital through intraperitoneal injection and placed in a supine position. Occlusion was achieved by placing a 50 g aneurysm clip above the right renal artery near the heart for 60 min. The SCIRI model was successfully established if neurological deficits appeared in the hindlimb. Bladder emptying was manually performed twice daily during the experimental period. Then, mice were randomly assigned into five groups (n = 8 per group); the Sham group, SCIRI group, sEVs group, Lenti-Ctrl-sEVs group, and Lenti-TSG-6-sEVs group. The SCIRI group and the other group were injected through the tail vein of PBS (200 µL), sEVs, Lenti-Ctrl-sEVs, or Lenti-TSG-6-sEVs (1 × 10^10^ particles of sEVs, Lenti-Ctrl-sEVs, or Lenti-TSG-6-sEVs per 200 µL) immediately after SCIRI.

### Functional locomotor scores

From the first postoperative day, neurological functions of mice were analyzed at regular time points using the Basso Mouse Scale (BMS) as previously described [37]. The BMS ranged from 0 points, indicating complete paralysis, to 9 points, indicating normal motor functions. The inclined plane test was also performed using a testing apparatus to evaluate locomotor functions in accordance with previous studies [38]. The maximum angle at which the mice could maintain its position for 5 s without falling was recorded. Behavioral assessments were performed by two trained observers who were blinded to the experiment.

### Footprint analysis

Footprint analysis was performed to evaluate gait and motor coordination of mice 4 weeks after reperfusion. Front and rear paws were coated with blue and red dyes, respectively. Then, mice were encouraged to walk in a straight line on white paper. The obtained footprint patterns were analyzed to assess the recovery of coordination ability.

### Swimming test

Motor functions were also evaluated using the Louisville Swim Score (LSS) 4 weeks post-injury. Louisville Swim Scale (LSS) was measured based on three swimming characteristics, including hindlimb alternation, forelimb dependency, and body position [39]. This trial was performed in duplicate.

### Preparation of spinal cord slices

After euthanasia, mice were transcardially perfused with 0.9% normal saline and 4% paraformaldehyde. The spinal cord was dissected out, fixed in 4% paraformaldehyde for 24 h, and sequentially dehydrated in 20% and 30% sucrose solutions. The tissue samples were embedded in optimal cutting temperature (OCT) and sliced into 10-μm longitudinal sections in a cryostat microtome (Leica, Germany).

### Measurement of blood–spinal cord barrier permeability

The disruption of BSCB was evaluated with Evan’s Blue dye extravasation as described previously [3, 4]. Briefly, 1 h after the intravenous injection of 2% EB dye (10 mg/Kg), mice were perfused with saline. Spinal cord tissues were excised, immersed in methanamide for 24 h at 60 °C, and centrifuged. Using a spectrophotometer (Bio-Rad, Hercules, USA), the optical density of supernatant was recorded. The concentration of EB was calculated as μg/g of spinal cord from a standard curve. Frozen sections were prepared and analyzed with a fluorescence microscope to determine the integrated optical density (IOD) of EB in spinal cord.

### Determination of apoptosis in spinal cord sections with TUNEL assay

The TUNEL assay was conducted following the manufacturer’s protocols. After fixation with 4% PFA, the frozen spinal cord sections were co-stained with the TUNEL reaction solution and DAPI. The images of TUNEL-positive cells were acquired with a fluorescence microscope.

### Immunofluorescence analysis

For immunofluorescence staining, cells or spinal cord sections were fixed in 4% PFA for 15 min and subsequently permeabilized in 0.2% Triton X-100/PBS for 20 min. The samples were treated with 5% bovine serum albumin (BSA) for 1 h at room temperature to block nonspecific binding. The samples were incubated overnight at 4 °C with primary antibody specific for rabbit anti-ZO1 (1:50, Invitrogen), rabbit anti-claudin-5 (1:100; Invitrogen), rabbit anti-occludin (1:50; Abcam), mouse anti-CD31 (1:200, Invitrogen), mouse anti-NeuN (1:500, Abcam), and rabbit anti-GRP78 (1:200, Proteintech). Samples were washed thrice with PBS and incubated with Alexa 594- or Alexa 488-conjugated secondary antibodies (1:200, Jackson ImmunoResearch, USA) for 1 h in the dark. Finally, nuclei were labeled with DAPI and images were obtained under similar exposure time and conditions.

### Western blot analysis

Proteins were isolated from cells and spinal cord tissues and quantified using the BCA assay. The protein samples were loaded on SDS–PAGE and transferred to a polyvinylidene difluoride (PVDF) membrane. Then, the membranes were blocked in 5% BSA for 1 h at room temperature and then treated with primary antibodies (Table 1). After washing with Tris-buffered saline with Tween (TBST), the membranes were incubated with HRP-conjugated secondary antibodies (1:2000, Thermo Fisher Scientific) for 1 h at room temperature. The protein bands were visualized using Western blotting detection reagents (ECL, Beyotime Institute of Biotechnology) and normalized to the expression level of GAPDH.

**Table 1:** Primary antibodies used for Western blot analysis

Antibodies	Source	Identifier
Caspase-3	Cell Signal Technology	Cat#9662
p-AKT	Cell Signal Technology	Cat #4060
Bcl-2	Abcam	Cat#ab182858
Bax	Abcam	Cat#ab32503
occludin	Abcam	Cat#ab216327
p-PERK	Abcam	Cat#ab229912
PERK	Abcam	Cat#ab229912
ATF6	Abcam	Cat#ab37149
p-IRE1α	Abcam	Cat#ab48187
IRE1α	Abcam	Cat#ab37073
GAPDH	Abcam	Cat#ab8245
ZO-1	Invitrogen	Cat #61–7300
claudin-5	Invitrogen	Cat #352,588
GRP78	Proteintech	Cat#11,587–1-AP
GRP94	Proteintech	Cat#14,700–1-AP

### Statistical analyses

Data are shown as mean ± standard deviation of at least three independent experiments. Differences between groups were compared with the Student’s t test and one-way ANOVA. All analyses were conducted using GraphPad 8.0.2 software (La Jolla, CA, USA). *P* values less than 0.05 (*p* < 0.05) were considered statistically significant.

## Results

### Identification of ADSCs-sEVs

ADSC-sEVs were isolated and purified from ADSC culture medium through differential centrifugation after which transmission electron microscopy (TEM), nanoparticle trafficking analysis (NTA), and Western blotting were performed to characterize their morphology, size distribution, and surface markers, respectively. NTA showed that particle size was about 80 nm in diameter (Additional file 1: Fig. S1a). The typical morphology of sEVs was confirmed by TEM (Additional file Additional file 1: Fig. S1b). Furthermore, the level of surface protein markers, including CD63, CD9, and CD81, was much higher in sEVs than cells. Moreover, Calnexin was not detected in sEVs (Additional file Additional file 1: Fig. S1c). Collectively, these data confirm that the nanoparticles were sEVs.

### ADSC-sEVs are taken up by neurons and inhibit apoptosis in vitro

**Fig. 1:**
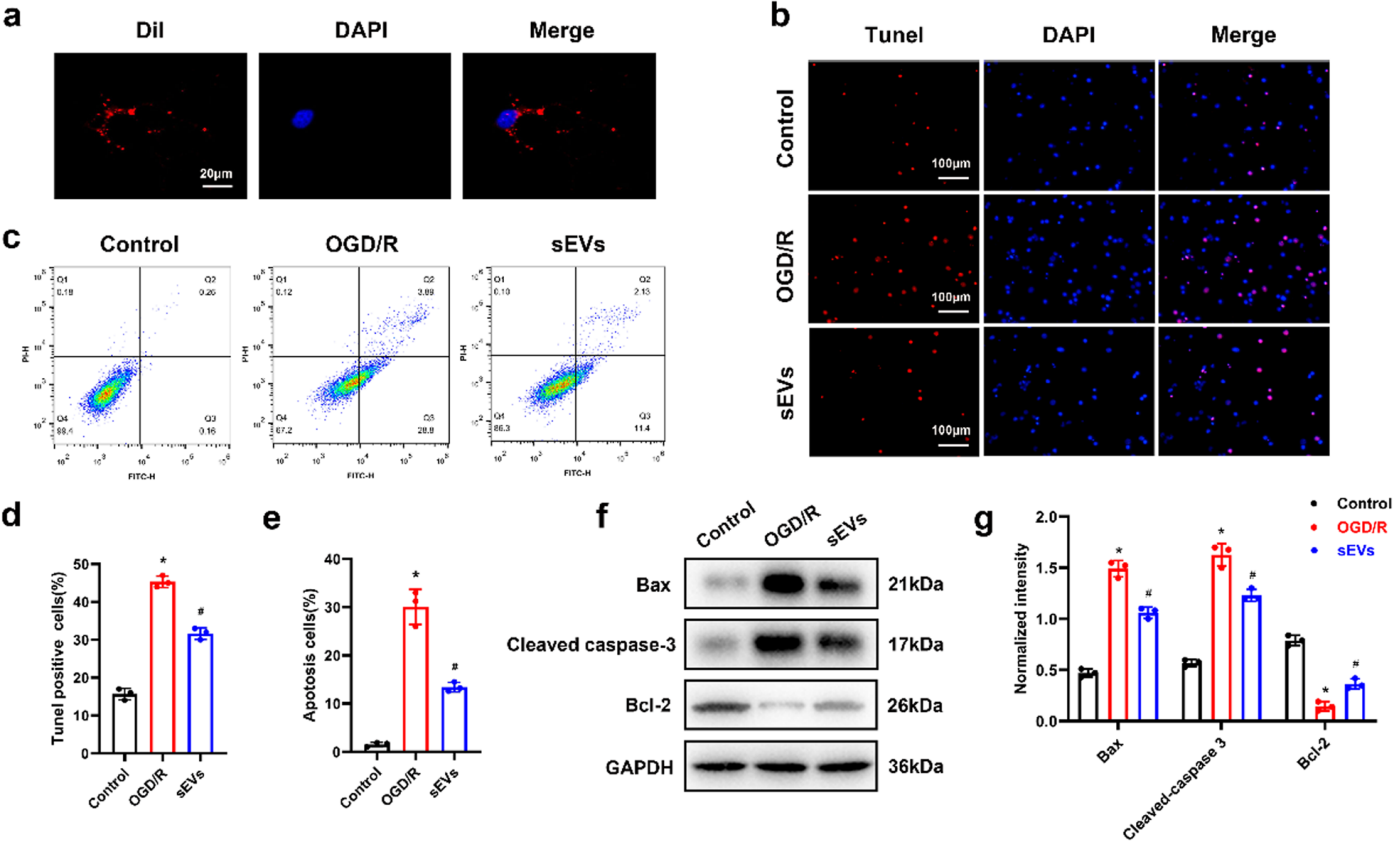
ADSC-sEVs were internalized by neurons to inhibit apoptosis in vitro. **a** Representative images of neurons incubated with Dil-labeled sEVs. The nuclei of neuron were stained with DAPI (blue). **b** TUNEL staining (red) showing apoptotic rate of primary neurons. Cell nuclei were counterstained with DAPI (blue). **c** Annexin V-FITC/PI double staining was determined by flow cytometry. **d** Quantitative estimation of the apoptotic rate in each group. ADSC-sEVs significantly reduced OGD/R-induced apoptosis. **e** Quantitative estimation of apoptosis rate of neurons with flow cytometry. **f** Expression of apoptosis-related proteins as measured by Western blotting analysis. **g** Relative expression levels of apoptosis-related proteins normalized to GAPDH. *p < 0.05 compared with the control group, ^#^*p* < 0.05 compared with the OGD/R group

To evaluate the neuroprotective effects of ADSC-sEVs in vitro, we first examined the internalization of labeled sEVs (DiI-sEVs). After incubating the neurons in the presence of Dil-sEVs for 24 h, sEVs uptake was detected using a confocal microscope. As shown in Fig. 1a, fluorescence-labeled sEVs were observed in the cytoplasm, indicating their internalization by neurons. TUNEL staining showed that oxygen–glucose deprivation and reperfusion (OGD/R) induced a relative increase in apoptotic ratio (TUNEL positive to total cells), which was significantly ameliorated by 24 h of pretreatment with ADSC-sEVs, indicating the neuroprotective effects of sEVs against OGD/R-induced apoptosis in primary neurons (Fig. 1b, d). In addition, cell apoptosis was evaluated by flow cytometry after Annexin V-FITC/PI double staining. Neuronal apoptosis was inhibited in OGD/R-treated cells supplemented with ADSC-sEVs (Fig. 1c, e).

Compared to the OGD/R group, pretreatment with ADSC-sEVs significantly promoted the expression of Bcl-2 (an anti-apoptotic protein) and downregulated the levels of pro-apoptotic proteins (Bax and cleaved caspase-3) (Fig. 1f, g).

### ADSC-sEVs inhibited the loss of tight junction proteins in bEnd.3 cells after OGD/R

**Fig. 2:**
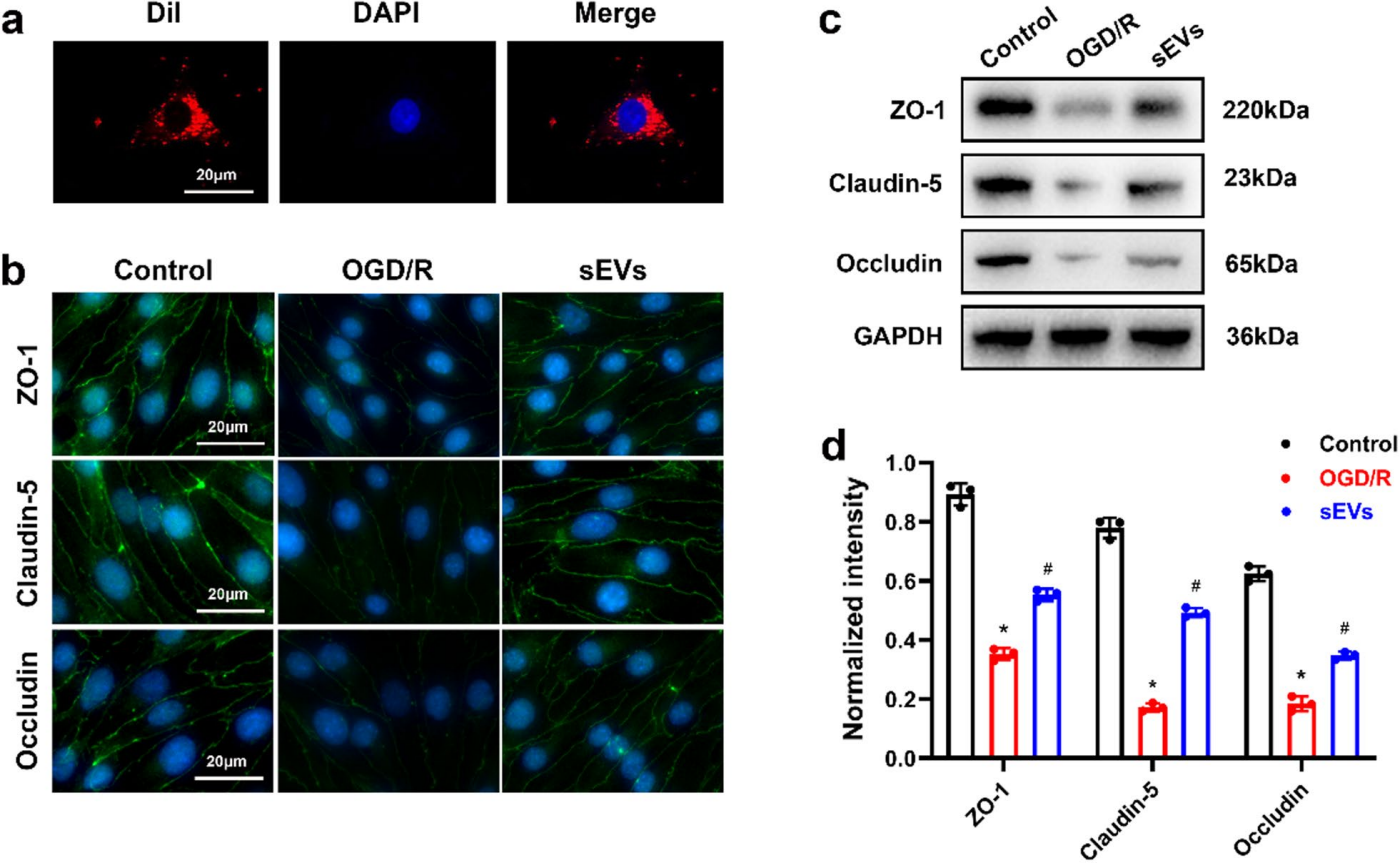
ADSC-sEVs inhibited loss of tight junction proteins in endothelial cells. **a** The Dil-sEVs were internalized by bEnd.3 cells. The nuclei of bEnd.3 cells were stained with DAPI (blue). **b** Representative immunofluorescence images of tight junction proteins. **c** Expression of tight junction proteins as determined by Western blotting analysis. **d** Relative expression levels of tight junction proteins normalized to GAPDH. *p < 0.05 compared with the control group, ^#^*p* < 0.05 compared with the OGD/R group

ADSC-sEVs were fluorescently labeled with Dil, and these nano-sized vesicles were taken up by bEnd.3 cells (Fig. 2a). Since tight junction proteins, including ZO-1, claudin-5, and occludin, maintain BSCB integrity, barrier function disruption is correlated with the degradation of these proteins. We evaluated whether pretreatment with ADSC-sEVs affected the expression of tight junction proteins in endothelial cells after OGD/R. As shown in Fig. 2b, OGD/R-induced down-regulation of tight junction protein expression was suppressed by pretreatment with ADSC-sEVs. Western blot also showed that compared to the OGD/R group, the loss of tight junction proteins was reversed by ADSC-sEVs (Fig. 2c, d).

### ADSC-sEVs treatment improved functional recovery and attenuated neuronal cell death following SCIRI

**Fig. 3:**
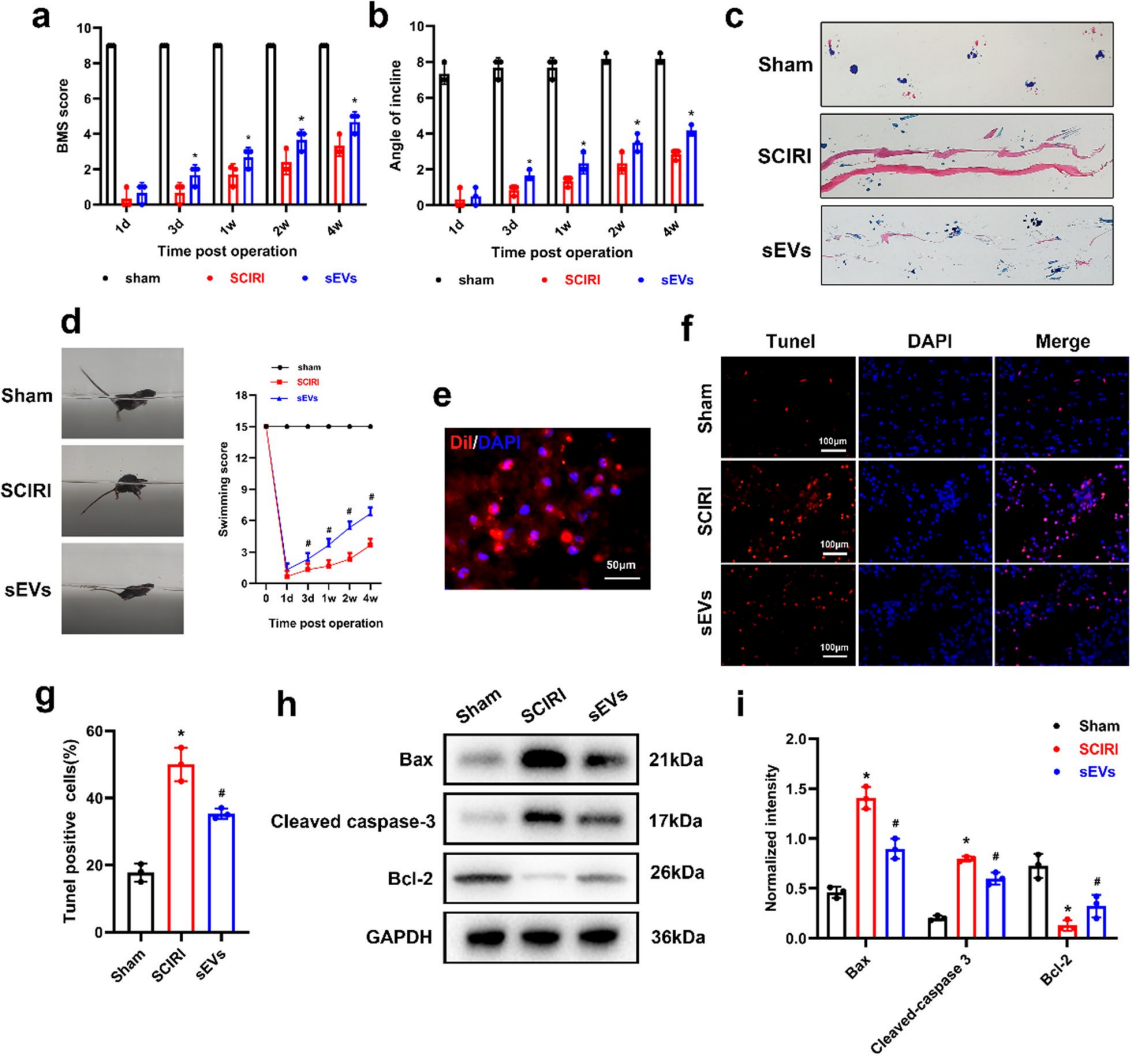
ADSC-sEVs treatment improved functional recovery and attenuated neuronal apoptosis following SCIRI. **a** Basso Mouse Scale (BMS) values at different times after SCIRI. **b** The inclined plane test at different times after SCIRI. **c** Representative images of the footprint assay performed 28 days after SCIRI. **d** Photographs of the swimming test conducted 28 days after SCIRI and statistical analysis of the Louisville Swim Scale. **e** Dil-sEVs accumulated in the injury area of spinal cord. **f** Analysis of apoptosis of neurons following SCIRI with the TUNEL assay. **g** Quantification of apoptosis rate in each experimental group. **h** Expression of apoptosis-related proteins following SCIRI as determined using Western blotting assay. **i** Relative expression levels of apoptosis-related proteins normalized to GAPDH. *p < 0.05 compared with the Sham group, ^#^*p* < 0.05 compared with the SCIRI group

To evaluate the neuroprotective effects of ADSC-sEVs on SCIRI, we established an animal model of SCIRI. BMS scores and inclined plate test showed that intravenously injected ASDC-sEVs group exhibited better motor function recovery than mice in the SCIRI group at 3, 7, 14, and 28 days post-operation (Fig. 3a, b). In gait analysis, hindlimb locomotor functions in the SCIRI group were significantly suppressed, while ADSC-sEVs treatment promoted gait and motor coordination recoveries (Fig. 3c). In addition, compared to mice in the SCIRI group, ADSC-sEVs treatment significantly improved Louisville Swim Scores (LSS) (Fig. 3d). In vivo experiments were performed to further investigate ADSC-sEVs uptake through systemic administration. As shown in Fig. 3e, Dil-sEVs can cross the blood–brain barrier and accumulate in the injured area of the spinal cord. TUNEL staining revealed that the rate of apoptotic cells was increased after OGD/R and was significantly reduced when treated with ADSC-sEVs (Fig. 3f, g). Compared to the SCIRI group, the expression levels of anti-apoptotic protein (Bcl-2) were significantly elevated, whereas the expression levels of apoptosis-related proteins (Bax and cleaved caspase-3) were significantly suppressed after ADSC-sEVs treatment (Fig. 3h, i). Collectively, these results imply that ADSC-sEVs treatment improved functional recovery and attenuated neuronal apoptosis following SCIRI.

### ADSC-sEVs treatment inhibited BSCB disruption following SCIRI

**Fig. 4:**
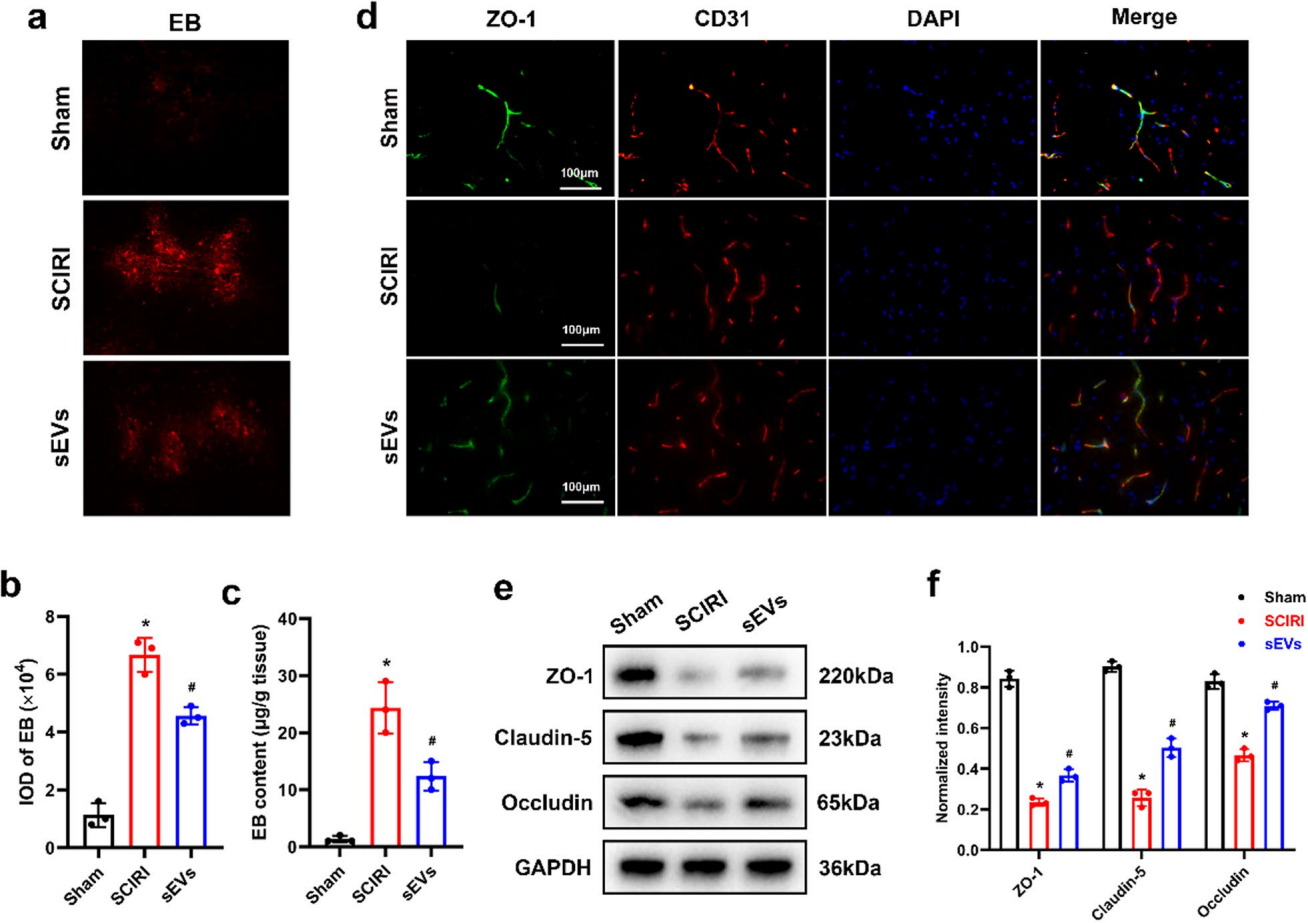
ADSC-sEVs treatment inhibited the disruption of BSCB following SCIRI. **a** Representative images of EB extravasation in the spinal cord sections. **b** Quantification of the intensity of Evans blue. The fluorescence intensity of EB is represented as the average integrated optical density (IOD). **c** Quantification of the amount of Evans blue in the spinal cord tissues. **d** Representative images showing double immunofluorescence of ZO-1 and CD31. **e** Expression of tight junction proteins in each experimental group was determined by Western blotting assay. **f** Relative expression levels of tight junction proteins normalized to GAPDH. *p < 0.05 compared with the Sham group, ^#^*p* < 0.05 compared with the SCIRI group

To evaluate the effect of ADSC-sEVs treatment on BSCB after SCIRI, EB extravasation contents in spinal cord samples were qualitatively and quantitatively assessed. As shown in Fig. 4a, almost no red fluorescence was detected in the Sham group. However, EB fluorescence intensity was higher in the SCIRI group than in the Sham group, and fluorescence intensity was significantly low after ADSC-sEVs treatment (Fig. 4a, b). Quantitatively, ADSC-sEVs significantly inhibited EB extravasation in the spinal cord tissues when compared to the SCRI group (Fig. 4c). Next, we evaluated SCIRI-induced tight junction protein alterations and the effect of ADSC-sEVs on the expression levels of these proteins. Double labeling immunofluorescence showed that the fluorescence intensity of CD31 and tight junction proteins, including ZO-1, claudin-5, and occludin, was decreased following SCIRI, while ADSC-sEVs treatment significantly attenuated the decrease in intensity (Fig. 4d, Additional file 2: Fig. S2a, b). Moreover, SCIRI-induced inhibition of the expression levels of tight junction proteins was significantly reversed after ADCS-sEVs treatment (Fig. 4e, f). These results show that ADSC-sEVs treatment prevents BSCB disruption after SCRI.

### ADSC-sEVs inhibited OGD/R-induced ER stress in vitro

Since ER is a central cellular organelle, and persistent ER stress can lead to cellular apoptosis, we evaluated the effect of ADSC-sEVs on ER stress in vitro. Immunofluorescence revealed that GRP78-labeled neurons were elevated in the OGD/R group and significantly decreased after ADSC-sEVs treatment (Additional file 3: Fig. S3a, b). Western blot analysis was used to establish the expression levels of three critical protein markers associated with UPR, including protein kinase-like endoplasmic reticulum kinase (PERK), inositol-requiring protein 1α (IRE1α), and activating transcription factor 6 (ATF6). The expression levels of ER stress–related proteins were significantly elevated in the OGD/R group compared to the control group, and this increase was significantly suppressed after ADSC-sEVs treatment (Additional file 3: Fig. S3c, d,e).

### ADSC-sEVs treatment suppressed ER stress following SCIRI

**Fig. 5:**
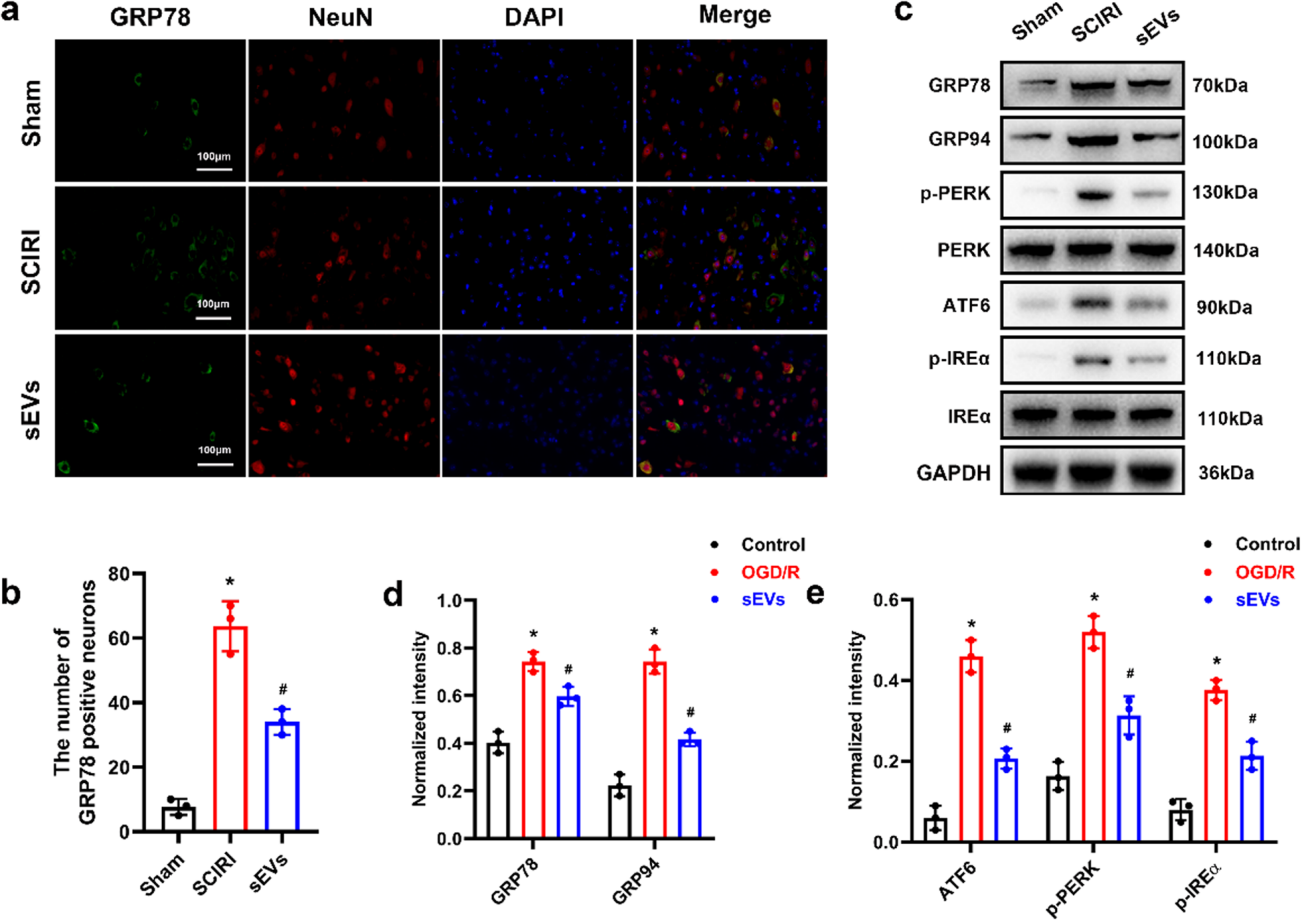
ADSC-sEVs treatment inhibited ER stress after SCIRI. **a** Representative images showing GRP78 expression in each experimental group. The nuclei of neurons were stained with DAPI. **b** Quantitative analysis of GRP78-positive neurons. **c** Expression of ER stress-related proteins as measured with Western blotting analysis. **d**, **e** Relative expression levels of ER stress-related proteins normalized to GAPDH. *p < 0.05 compared with the Sham group, ^#^*p* < 0.05 compared with the SCIRI group

The endoplasmic reticulum is an important cellular organelle that is involved in protein synthesis and persistent ER stress without treatment activates cell injury. Given the crucial role of ER stress in the pathological mechanisms of SCIRI, we evaluated the effect of ADSC-sEVs on ER stress following SCIRI. Compared to the Sham group, the expression levels of GRP78-positive neurons increased after SCIRI, and intravenous injection of ADSC-sEVs significantly inhibited the GRP78 expression levels (Fig. 5a, b). Furthermore, the expression levels of proteins associated with UPR and ER stress were significantly upregulated following SCIRI. However, ADSC-sEVs treatment significantly suppressed the expression of these proteins (Fig. 5c, d, e). These results show that ADSC-sEVs treatment inhibited SCIRI-induced ER stress.

### ADSC-sEVs inhibit neuronal apoptosis and BSCB disruption by suppressing ER stress

**Fig. 6:**
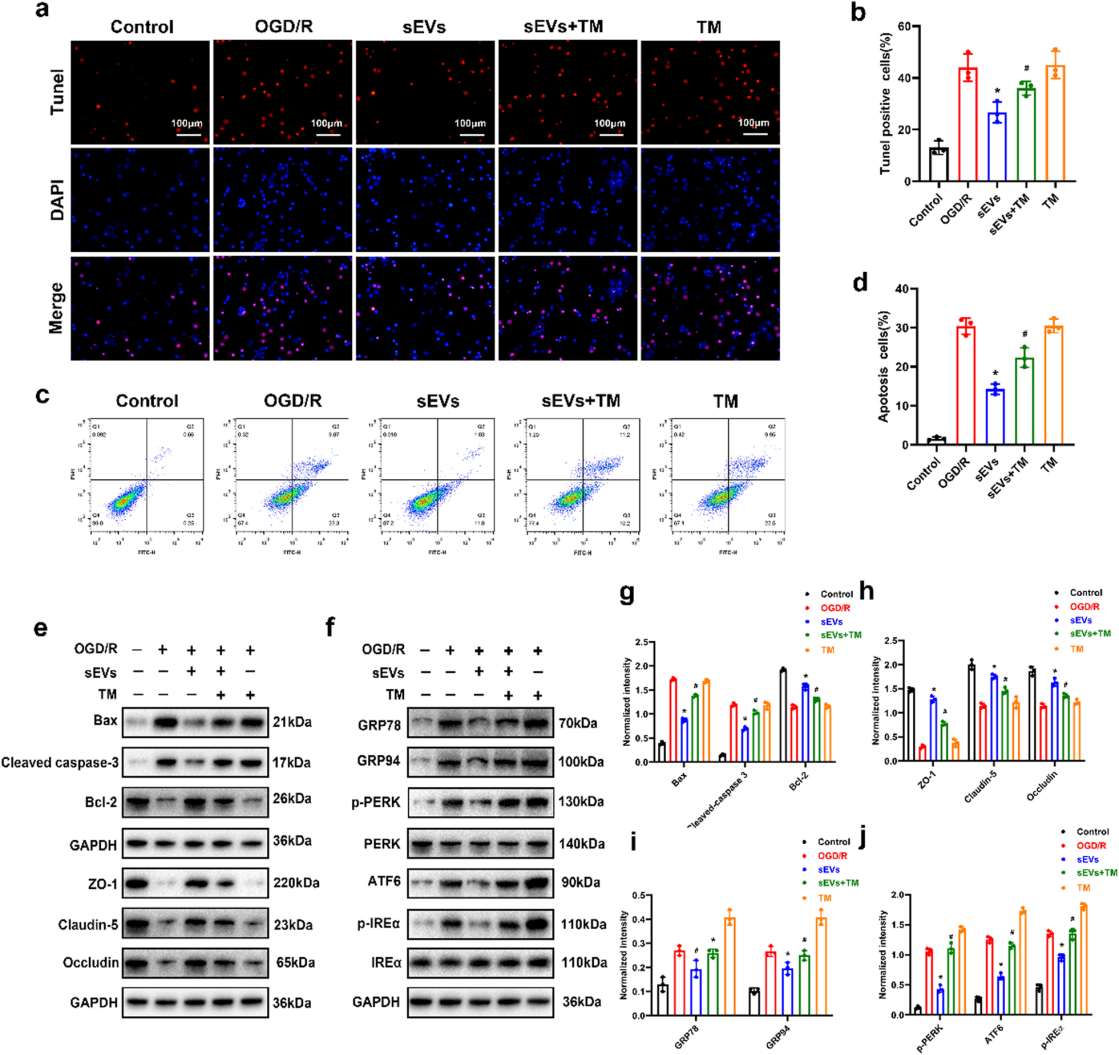
ADSC-sEVs reduce apoptosis and BSCB disruption by suppressing ER stress. **a** Results of the TUNEL assay showing the number of apoptotic neurons induced by OGD/R with or without ADSC-sEVs and TM pretreatment. **b** Quantitative estimation of the apoptotic rate in each group. **c** Results of Annexin V-FITC/PI double staining as analyzed by flow cytometry. **d** Quantitative estimation of the apoptotic rate by flow cytometry. **e** Expression of apoptosis-related and tight junction proteins as determined by Western blotting analysis. **f** Western blotting analysis of ER stress-related proteins. **g**–**j** Relative expression levels of apoptosis, ER stress-related proteins, and tight junction proteins normalized to GAPDH. *p < 0.05 compared with the OGD/R group, ^#^*p* < 0.05 compared with the ADSC-sEVs group

Given the essential role of ER stress in neuronal apoptosis and BSCB integrity, we postulated that inhibition of neuronal apoptosis and BSCB disruption following ADSC-sEVs treatment depend on suppression of ER stress [40, 41]. To confirm this hypothesis, primary neurons were treated with ADSC-sEVs in the presence or absence of the ER stress inducer, tunicamycin (TM), prior to OGD/R. The OGD/R-induced increase in apoptotic ratio was significantly ameliorated by ADSC-sEVs treatment (Fig. 6a, b). However, the anti-apoptotic effect of ADSC-sEVs was partially reversed by the ER stress inducer, tunicamycin (Fig. 6a, b). Findings from flow cytometry with Annexin V-FITC/PI and Western blot assays were consistent with those of TUNEL staining (Fig. 6c, d, e, g). Furthermore, compared to the ADSC-sEVs treated group, Western blot showed that TM upregulated the expression of ER stress-related proteins (Fig. 6i, j). Finally, TM inhibited ADSC-sEVs-induced elevation in the expression of tight junction proteins (Fig. 6e, h). In summary, ADSC-sEVs exert its anti-apoptotic effect and protect BSCB integrity by suppressing ER stress.

### ADSC-sEVs transport tumor necrosis factor (TNF)-stimulated gene-6 (TSG-6) into spinal cord tissues and overexpression of TSG-6 enhances the repair of sEVs in motor function after SCIRI

TSG-6, a multifunctional glycoprotein, has been shown to down-regulate ER stress and exerting anti-inflammatory effects in a variety of diseases, including acute pancreatitis, brain injury, inflammatory bowel disease, and osteoarthritis [42–45]. However, it has not been established whether ADSC-sEVs associated with TSG-6 play a crucial role in SCIRI repair.

**Fig. 7:**
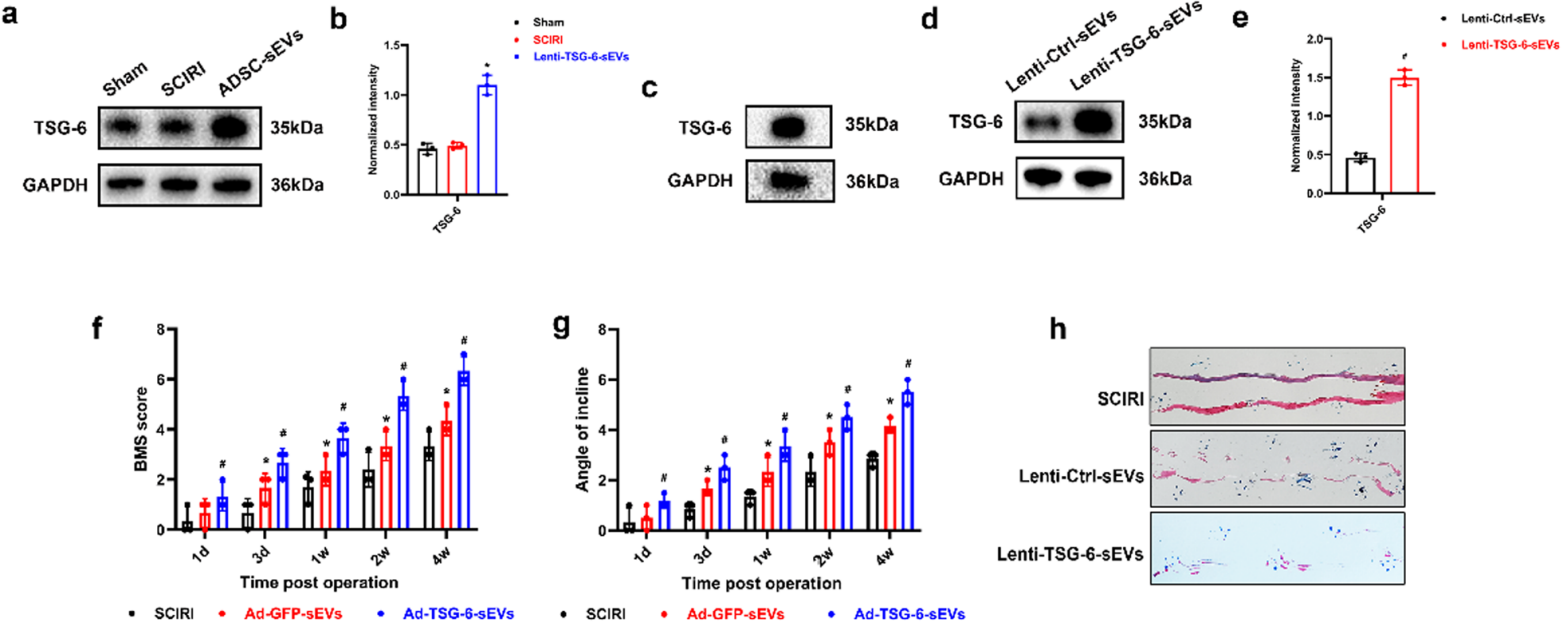
ADSC-sEVs transported TSG-6 proteins into spinal cord tissue and overexpression of TSG-6 enhanced the capacity of sEVs to improve motor function after SCIRI. **a**. Expression of TSG-6 expression in spinal cord tissue after ADSC-sEVs administration as determined by Western blotting analysis. **b** Relative expression levels of TSG-6 in spinal cord tissues, normalized to GAPDH. **c** Expression of TSG-6 in ADSC-sEVs as determined by Western blotting assay. **d**, **e** Determination of TSG-6 expression in Lenti-TSG-6-sEVs and Lenti-Ctrl-sEVs by Western blotting assay. **f**, **g** Values of Basso Mouse Scale (BMS) and inclined plane test at different times after SCIRI. **h** Representative images of the footprint assay performed 28 days after SCIRI. *p < 0.05 compared with the SCIRI group, ^#^*p* < 0.05 compared with the Lenti-Ctrl-sEVs group

In this study, LCMS/MS analysis revealed that ADSC-sEVs contain abundant TSG-6. Western blot analysis showed that, compared to the Sham and SCIRI groups, ADSC-sEVs significantly upregulated the expression of TSG-6 proteins in spinal cord tissues (Fig. 7a, b). Furthermore, Fig. 7c shows that ADSC-sEVs encapsulated the TSG-6 proteins, implying that ADSC-sEVs can transport TSG-6 into the spinal cord to perform various biological functions following SCIRI. To evaluate the protective effects of TSG-6 in vivo, we overexpressed TSG-6 in ADSCs through lentivirus transfection (Lenti-TSG-6), while an empty vector construct was used as a negative control (Lenti-Ctrl). TSG-6 overexpression was successfully achieved and confirmed by Western blot (Fig. 7d, e). BMS scores and inclined plate test revealed that mice in the Lenti-Ctrl-sEVs group and in the Lenti-TSG-6-sEVs group exhibited significant motor function recoveries. However, improvement in the Lenti-TSG-6-sEVs group was more obvious (Fig. 7f, g). Footprint analysis also showed that mice in the Lenti-TSG-6-sEVs group exhibited better motor function recoveries than mice in the Lenti-Ctrl-sEVs group (Fig. 7h).

### Overexpression of TSG-6 enhances the anti-apoptotic and BSCB protection effect following SCIRI

**Fig. 8:**
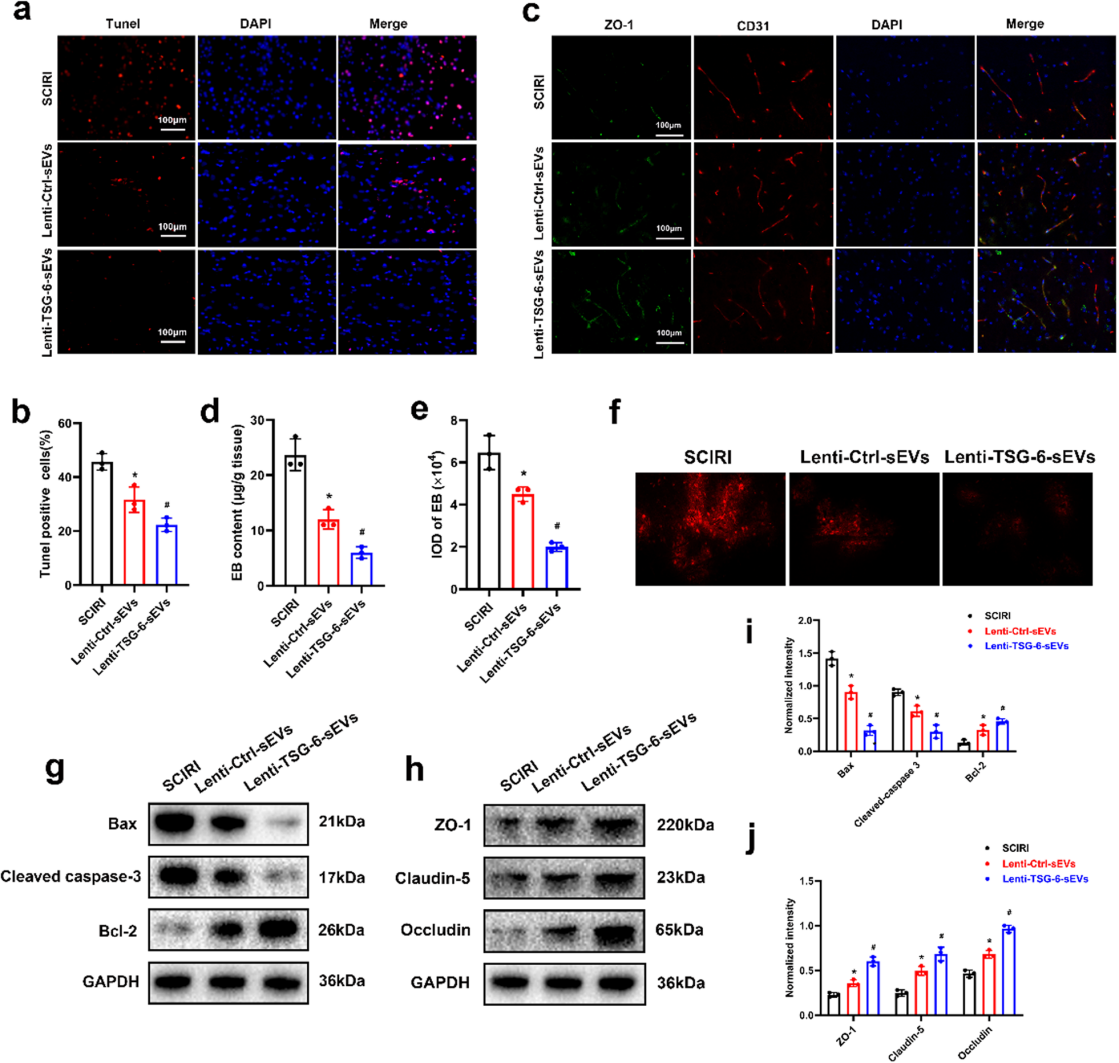
Overexpression of TSG-6 enhanced the protective effect of ADSC-sEVs following SCIRI. **a** TUNEL staining was conducted to measure apoptosis rate in each group. **b** Quantitative estimation of the apoptotic rate in each group. **c** Representative images showing double immunofluorescence of ZO-1 and CD31. **d** Quantification of the content of Evans blue in the spinal cord tissues. **e** Quantification of the fluorescence intensity of Evans blue. **f** Representative images of EB extravasation in the spinal cord sections. **g**, **h** Expression of apoptosis-related and tight junction proteins in each experimental group as determined by Western blotting assay. **i**, **j** Relative expression levels of apoptosis-related and tight junction proteins normalized to GAPDH. *p < 0.05 compared with the SCIRI group, ^#^*p* < 0.05 compared with the Lenti-Ctrl-sEVs group

The number of TUNEL-positive cells after Lenti-Ctrl-sEVs treatment was significantly reduced compared to the SCIRI group, whereas treatment with Lenti-TSG-6-sEVs further suppressed apoptosis (Fig. 8 a, b). Findings from Western blot analysis were consistent with those of TUNEL staining (Fig. 8 g, i).

To further evaluate the effect of TSG-6 on BSCB protection, double labeling immunofluorescence was performed. Compared to the SCIRI group, the Lenti-Ctrl-sEVs treatment group showed increased fluorescence intensities of CD31 and tight junction proteins, including ZO-1, claudin-5, and occludin. However, the increase in fluorescence intensities was more pronounced in the Lenti-TSG-6-sEVs treatment group (Fig. 8c, Additional file 4: Fig. S4a, b). Quantitative and qualitative analysis of EB showed that Lenti-TSG-6-sEVs exhibited better protective effects on BSCB integrity (Fig. 8 d, e, f). In addition, Western blot analyses of spinal cord tissues showed that, compared to the Lenti-Ctrl-sEVs group, Lenti-TSG-6-sEVs elevated the expression levels of tight junction proteins (Fig. 8 h, j). In summary, the overexpression of TSG-6 enhanced the anti-apoptotic and BSCB protection effect following SCIRI.

### Overexpression of TSG-6 enhanced ER stress suppression by modulating the PI3K/AKT signaling pathway

**Fig. 9:**
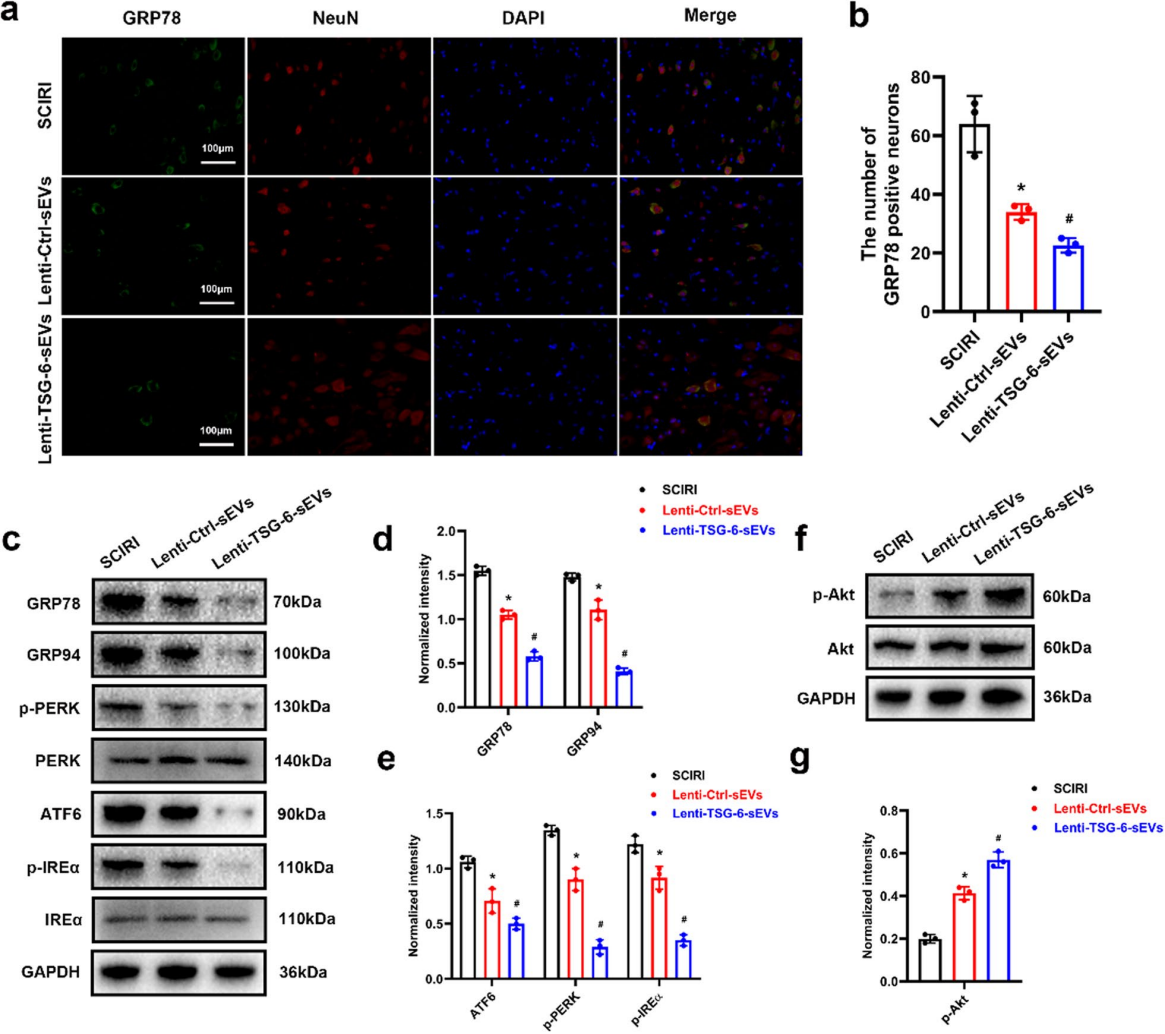
Overexpression of TSG-6 suppressed ER stress by modulating the PI3K/AKT signaling pathway. **a** Representative images of GRP78-positive neurons in each experimental group. **b** Quantitative analysis of GRP78-positive neurons. **c** Expression of ER stress-related proteins as determined by Western blotting analysis. **d**, **e** Relative expression levels of ER stress-related proteins normalized to GAPDH. **f** Western blot analysis of AKT protein. **g** Relative expression levels of p-AKT normalized to GAPDH. *p < 0.05 compared with the SCIRI group, ^#^*p* < 0.05 compared with the Lenti-Ctrl-sEVs group

It has been reported that mesenchymal stem cell-derived TSG-6 can significantly suppress ER stress-induced apoptosis [42]. We further determined whether TSG-6 overexpression enhances ER stress suppression in vivo. Figure 9 a, b shows that, compared to the SCIRI group, the number of GRP78-positive cells was significantly reduced in the Lenti-Ctrl-sEVs group, while Lenti-TSG-6-sEVs treatment further attenuated the expression of GRP78. After TSG-6 overexpression, changes in ER stress were also evaluated by Western blot and the results were consistent with those of immunofluorescence analysis (Fig. 9 c, d, e).

Next, we investigated the signaling molecules that mediate ER stress suppression. Since it has been reported that the PI3K/AKT pathway is a canonical signaling pathway involved in ER stress, further experiments were performed to determine whether the effects of ADSC-sEVs on ER stress are associated with PI3K/AKT pathway modulation. Western blot showed that Lenti-Ctrl-sEVs treatment upregulated the expression of p-AKT; moreover, the upregulation was successfully enhanced by Lenti-TSG-6-sEVs treatment (Fig. 9 f, g).

## Discussion

SCIRI is one of the most catastrophic complications of aortic aneurysm repair or spinal cord surgery. Currently, clinically effective therapeutic options for SCIRI are extremely limited. Due to the complex molecular cascades affecting neurological functions, the mechanisms underlying SCIRI pathogenesis have not been elucidated; however, it has been reported that neuronal apoptosis and BSCB disruption play crucial roles in this process. We established that ADSC-sEVs attenuate neuronal apoptosis and inhibit BSCB disruption by suppressing hyperactive ER stress. Furthermore, the TSG-6 secreted by ADSC-sEVs and PI3K/AKT signaling pathway is involved in the inhibition of ER stress.

Stem cell-based therapy is an emerging therapeutic modality for the treatment of central nervous diseases [46–48]. ADSCs are a subset of mesenchymal stem cells (MSCs) whose unique characteristics including easy availability and high abundance make them optimal MSC sources. In addition, ADSCs have a robust capacity to differentiate into multiple lineages, not only adipocytes but also osteoblasts, chondrocytes, and neuronal cells. However, clinical applications of stem cells confer a considerable risk for tumorigenesis and deformity [49].

Due to the paracrine effects of MSCs, a novel strategy to apply components of the MSC secretome as an effective therapy was therefore proposed. The secretome of MSC is the set of factors/molecules, including protein fraction and different subtypes of extracellular vesicles, secreted into the extracellular space. Classically, extracellular vesicles can be divided into three groups based on their biogenesis: apoptotic bodies, microvesicles, and small extracellular vesicles. Apoptotic bodies have the most heterogeneous size (200 nm-5 μm) and are secreted by a dying cell, while microvesicles (100 nm–800 nm) and small extracellular vesicles (30 nm–150 nm) are secreted by live cells. Small extracellular vesicles containing cell-type-specific combinations enhance the therapeutic efficacies of stem cells [50]. Therefore, we hypothesized that intravenous injection of ADSC-sEVs can overcome the limitations associated with stem cell transplantation and improve the neurological deficits following SCIRI.

To verify this hypothesis, a series of in vitro and in vivo experiments were performed. First, sEVs were successfully extracted from the culture medium of ADSCs and characterized by TEM, NTA, and Western blot. It has been documented that neuronal death is induced by apoptosis following ischemia–reperfusion injury; therefore, we evaluated the effects of ADSC-sEVs on neuronal apoptosis. Flow cytometry, TUNEL staining, and Western blot revealed that ADSC-sEVs treatment significantly inhibited OGD/R-induced neuronal apoptosis. To confirm these anti-apoptotic effects, TUNEL-positive neurons and apoptosis-related proteins were evaluated in spinal cord samples and in vivo results demonstrated that ADSC-sEVs effectively inhibited cell apoptosis after SCIRI.

The BSCB, which is a highly selective brain endothelial structure between the peripheral circulation and central nervous system, provides a particular homeostasis for the spinal cord [51]. Under different pathological conditions such as SCIRI, spinal cord injury, multiple sclerosis, and epilepsy, the integrity of BSCB is damaged and immune cells infiltrate into the spinal cord, contributing to secondary injuries. The tight junction complexes between brain endothelial cells are the key elements in BSCB, and their degradation enhances BSCB permeability [52]. A recent study demonstrated that administration of bone mesenchymal stem cell-derived extracellular vesicles (BMSC-EV) improved motor function and maintained the structural integrity of the blood–spinal cord barrier following traumatic spinal cord injury [6]. We assessed the effects of ADSC-sEVs in vitro and found that they significantly inhibited OGD/R-induced degradation of tight junction proteins. In this study, EB extravasation was significantly inhibited in the ADSC-sEVs group after SCIRI. In summary, ADSC-sEVs treatment improves the expression of tight junction proteins and inhibits BSCB disruption.

The endoplasmic reticulum is an important cellular organelle that is involved in protein synthesis and secretion in eukaryotic cells, which are essential for maintaining cellular homeostasis [53, 54]. Perturbation of ER homeostasis by ischemia–reperfusion injury leads to the accumulation of misfolded proteins in the ER lumen, and persistent ER stress without treatment activates cell injury [55]. In recent years, an increasing number of studies have focused on the treatment of SCIRI by attenuating ER stress-related pathology [56, 57]. Given the crucial role of ER stress in the pathological mechanisms of SCIRI, we evaluated the effect of ADSC-sEVs on ER stress. We found that ADSC-sEVs pretreatment suppressed OGD/R-induced ER stress in vitro, findings that were confirmed by Western blotting of ER stress-related proteins. Similar findings were observed in SCIRI animal models following ADSC-sEVs treatment. Studies have documented that ER stress is associated with neuronal apoptosis and disruption of BSCB integrity [40, 41, 58]. Pharmacological experiments have revealed that the effects of anti-apoptotic and inhibition of BSCB disruption are directly dependent on the modulation of ER stress.

TSG-6 is a multifunctional protein involved in various biological functions [28]. It has been shown that TSG-6 secreted by ADSC improves severe acute pancreatitis by suppressing ER stress [42]. In this study, LCMS/MS analysis revealed that ADSC-sEVs contain TSG-6, consistent with Western blot results. Besides, the expression of TSG-6 was upregulated in the ADSC-sEVs, indicating that TSG-6 was transported from sEVs into the spinal cord tissues after SCIRI. Additionally, overexpression of TSG-6 promoted the anti-apoptotic effect, BSCB protection, and enhanced the suppression of ER stress in vivo. PI3K/Akt signaling at the mitochondria-associated endoplasmic reticulum membranes plays crucial roles in various biological processes such as glucose homeostasis and cell proliferation. Previous studies confirmed that phosphorylation of PI3K and Akt inhibited ER stress in the lungs [59]. Activation of the PI3K/AKT pathway was also found to suppress ER stress-induced apoptosis [60]. We further evaluated the effect of ADSC-sEVs-associated TSG-6 on the PI3K/AKT pathway. The present study indicates that sEVs upregulated the expression of p-AKT and TSG-6 secreted by ADSC-sEVs-suppressed ER stress by modulating the PI3K/AKT pathway. Our studies provide further insight into sEVs-mediated effects on ER stress through PI3K/AKT pathway.

## Conclusions

In conclusion, ADSC-sEVs inhibit neuronal apoptosis and protect BCSB integrity after SCIRI by suppressing ER stress. Furthermore, this study also suggests that the inactive of excess ER stress was achieved by TSG-6 secreted by ADSC-sEVs through the modulation of PI3K/AKT pathway. These findings provide a new therapeutic strategy for the treatment of SCIRI.

## Supplementary Information

**Additional file 1** Figure S1: Characterization of ADSC-sEVs. **a** Particle size distribution of sEVs measured by nanoparticle trafficking analysis (NTA). **b** Typical morphology as detected by a transmission electron microscope (TEM). **c** Western blot results of specific surface markers. Figure S2 ADSC-sEVs treatment inhibited tight junction disruption following SCIRI. **a** Representative images showing double immunofluorescence of CD31 and Claudin-5. **b** Representative images showing double immunofluorescence of CD31 and occludin. Figure S3 ADSC-sEVs treatment suppressed ER stress in vitro. **a** Representative images showing GRP78 expression in each experimental group. The nuclei of neuron were stained with DAPI. **b** Quantitative analysis of fluorescence intensity of GRP78. **c** Expression of ER stress-related proteins as determined by Western blotting analysis. **d**, **e** Relative expression levels of ER stress-related proteins normalized to GAPDH. *p < 0.05 compared with the control group, #*p* < 0.05 compared with the OGD/R group. Figure S4 Overexpression of TSG-6 promoted the protective effect of ADSC-sEVs on the integrity of BSCB following SCIRI. **a** Representative images showing double immunofluorescence of CD31 and claudin-5. **b** Representative images showing double immunofluorescence of CD31 and occludin.

## Data Availability

Most of the datasets supporting the conclusions of this article are included within this article and the additional files. The datasets used or analyzed during the current study are available on reasonable request.

## Abbreviations

SCIRI: Spinal cord ischemia reperfusion injury
BSCB: Blood–spinal cord barrier
ZO-1: Zonula occludens-1
ADSC: Adipose‐derived stem cell
MeeSC: Mesenchymal stem cell
sEVs: Small extracellular vesicles
ER: Endoplasmic reticulum
UPR: Unfolded protein response
TEM: Transmission electron microscopy
NTA: Nanoparticle trafficking analysis
TUNEL: Terminal deoxynucleotidyl transferase dUTP nick end labeling
OGD/R: Oxygen–glucose deprivation and reperfusion
LSS: Louisville Swim Scores
TM: Tunicamycin
TSG-6: Tumor necrosis factor-stimulated gene-6
PBS: Phosphate-buffered saline
DMEM: Dulbecco’s modified Eagle’s medium
FBS: Fetal bovine serum
BMS: Basso Mouse Scale
OCT: Optimal cutting temperature
EB: Evans blue
