# Supplementary figures and images for: TSG-6 released from adipose stem cells-derived small extracellular vesicle protects against spinal cord ischemia reperfusion injury by inhibiting endoplasmic reticulum stress
Source: Stem Cell Res Ther. 2022 Jul 13;13:291. doi: 10.1186/s13287-022-02963-4 (PMC9281104; doi:10.1186/s13287-022-02963-4)

Figure S1


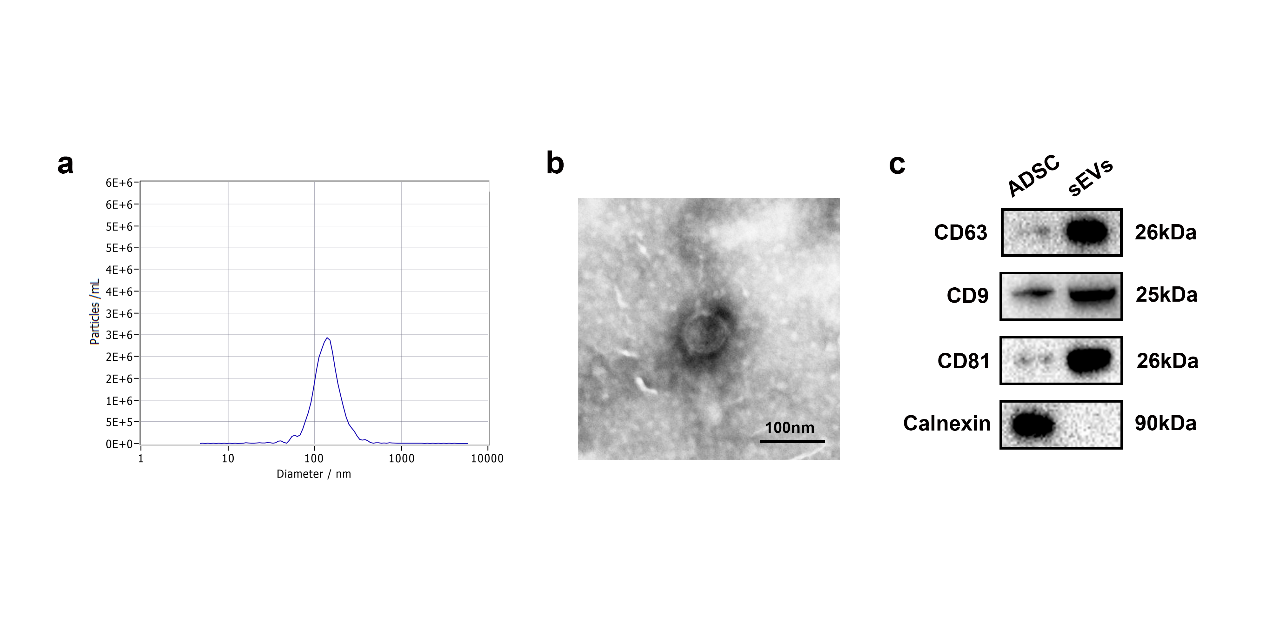


Figure S2


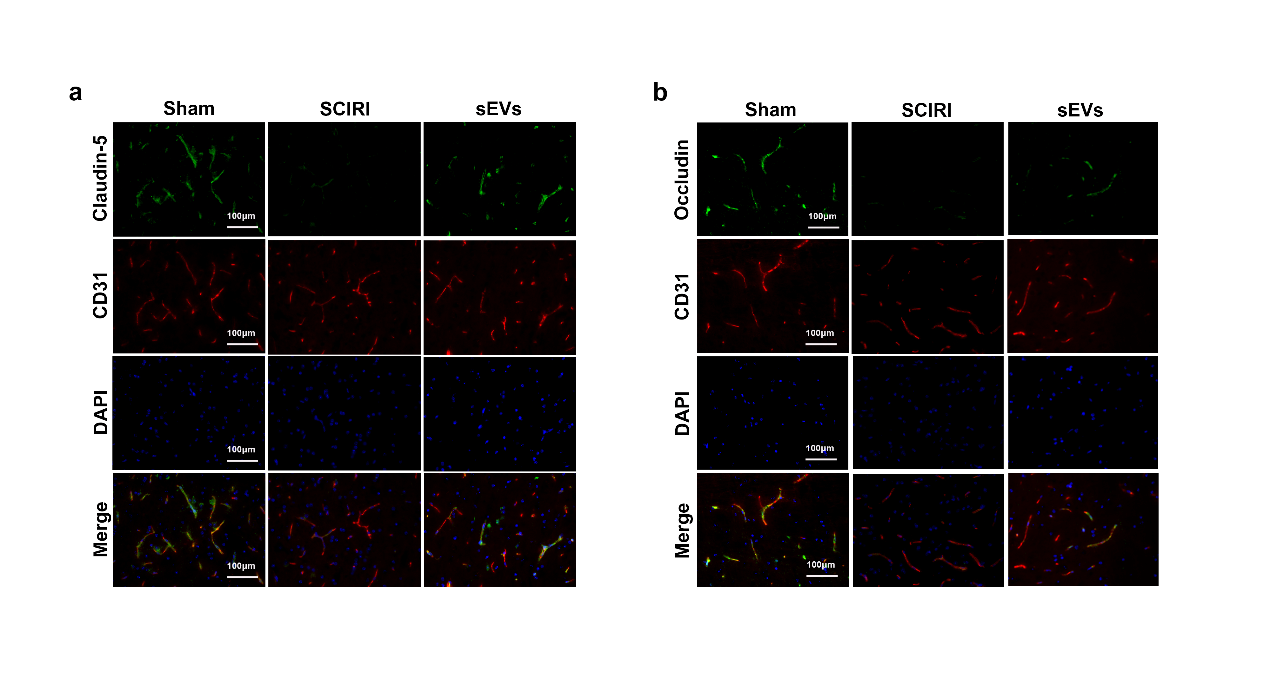


Figure S3


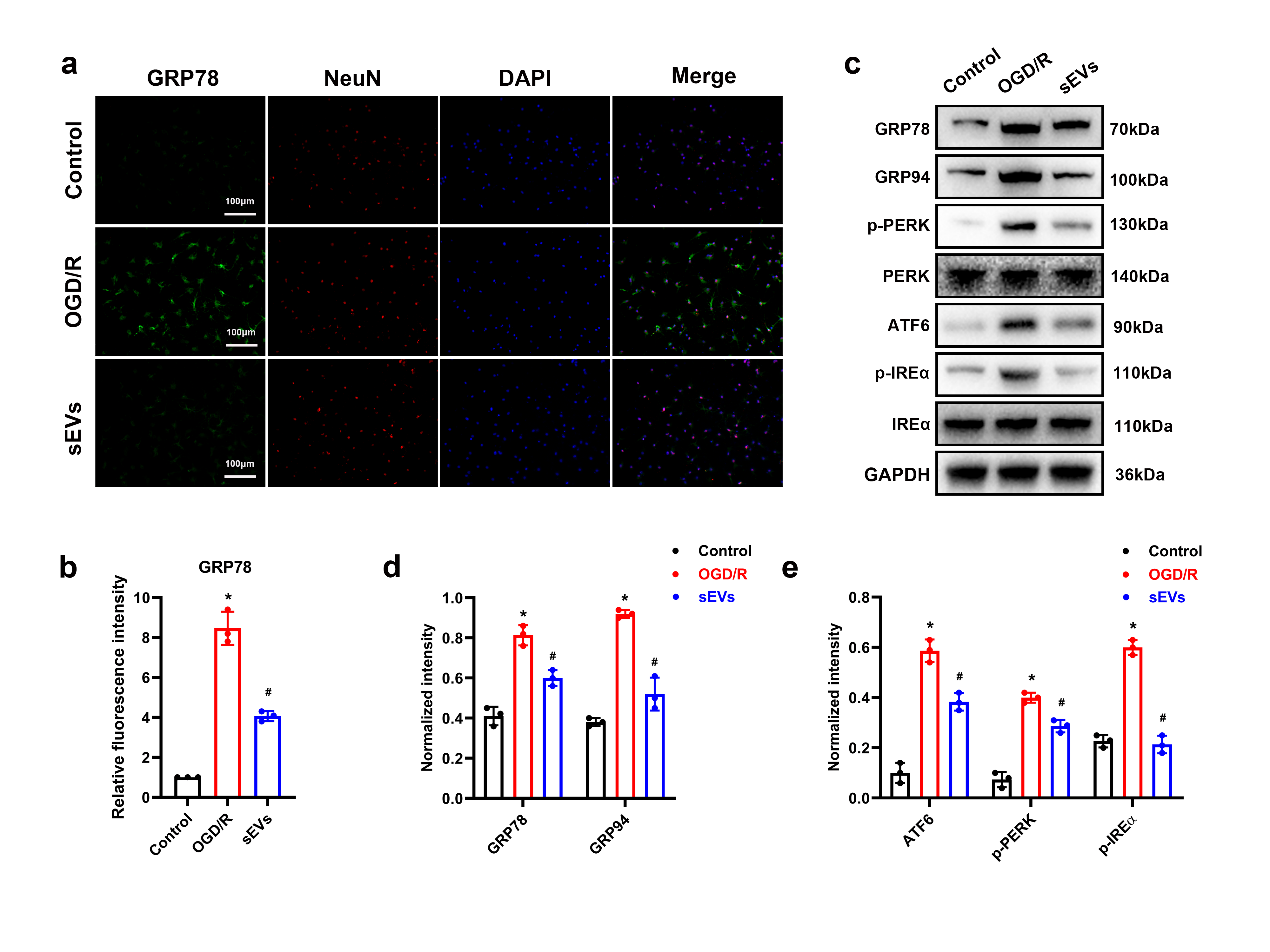


Figure S4


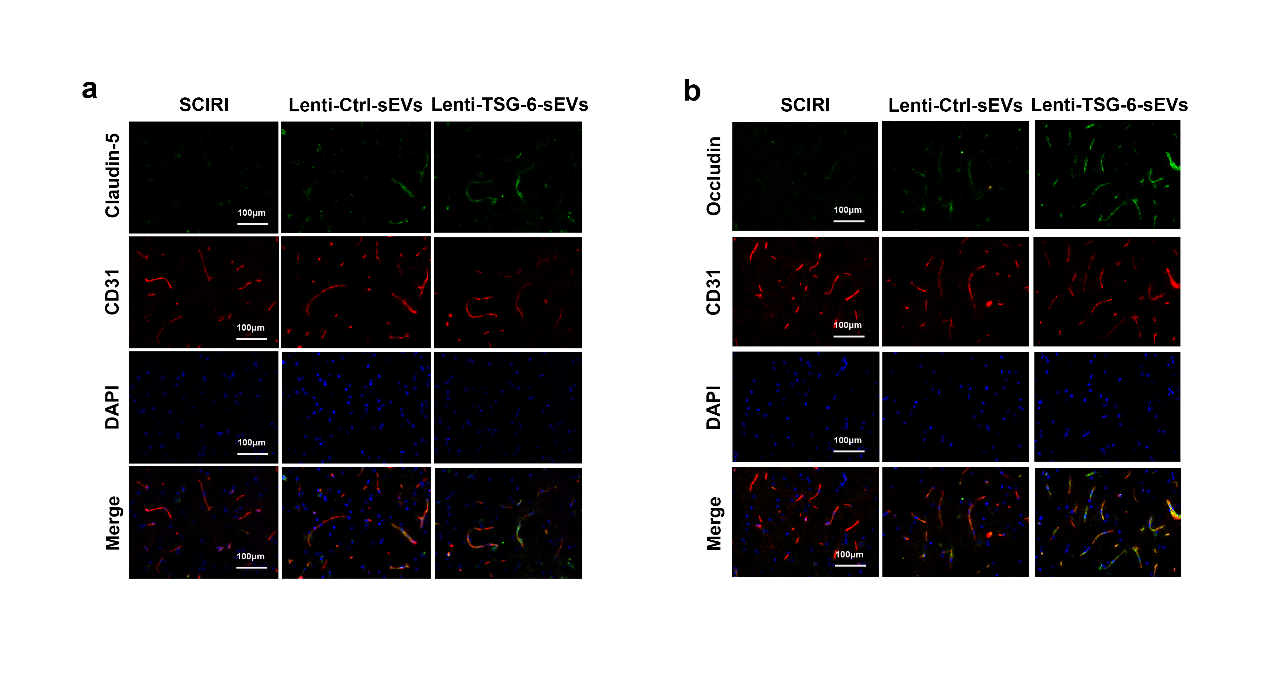

Supplement: Supplementary file 1 — Additional file 1 Figure S1: Characterization of ADSC-sEVs. a Particle size distribution of sEVs measured by nanoparticle trafficking analysis (NTA). b Typical morphology as detected by a transmission electron microscope (TEM). c Western blot results of specific surface markers. Figure S2 ADSC-sEVs treatment inhibited tight junction disruption following SCIRI. a Representative images showing double immunofluorescence of CD31 and Claudin-5. b Representative images showing double immunofluorescence of CD31 and occludin. Figure S3 ADSC-sEVs treatment suppressed ER stress in vitro. a Representative images showing GRP78 expression in each experimental group. The nuclei of neuron were stained with DAPI. b Quantitative analysis of fluorescence intensity of GRP78. c Expression of ER stress-related proteins as determined by Western blotting analysis. d, e Relative expression levels of ER stress-related proteins normalized to GAPDH. *p < 0.05 compared with the control group, #p < 0.05 compared with the OGD/R group. Figure S4 Overexpression of TSG-6 promoted the protective effect of ADSC-sEVs on the integrity of BSCB following SCIRI. a Representative images showing double immunofluorescence of CD31 and claudin-5. b Representative images showing double immunofluorescence of CD31 and occludin. [file 13287_2022_2963_MOESM1_ESM.docx]
